# Microstructure, Hot Deformation Behavior, and Recrystallization Behavior of Zn-1Fe-1Mg Alloy under Isothermal Compression

**DOI:** 10.3390/ma14071735

**Published:** 2021-04-01

**Authors:** Penghao Xue, Minglong Ma, Yongjun Li, Xinggang Li, Jiawei Yuan, Guoliang Shi, Kaikun Wang, Kui Zhang

**Affiliations:** 1 State Key Laboratory of Nonferrous Metals and Processes, GRIMAT Engineering Institute Co., Ltd., Beijing 100088, China; b20170187@xs.ustb.edu.cn (P.X.); maminglong@grinm.com (M.M.); lyj@grinm.com (Y.L.); lxg1218@grinm.com (X.L.); shigl@grinm.com (G.S.); 2 School of Materials Science and Engineering, University of Science & Technology Beijing, Beijing 100083, China; kkwang@mater.ustb.edu.cn; 3 GRIMAT Engineering Institute Co., Ltd., Beijing 101407, China; 4 General Research Institute for Nonferrous Metals, Beijing 100088, China

**Keywords:** biodegradable Zn-Fe-Mg, constitutive equation, twinning, dynamic recrystallization, microstructure evolution

## Abstract

Nowadays, wrought zinc-based biodegradable alloys are favored by researchers, due to their excellent mechanical properties and suitable degradation rates. However, there are few research studies on their thermal deformation behavior at present. This study took Zn-1Fe-1Mg and explored its microstructural change, deformation, recrystallization behavior and processing map by means of the thermal simulation experiment, at temperatures ranging from 235 °C to 340 °C and strain rates ranging from 10^−2^ s^−1^ to 10 s^−1^. The constitutive model was constructed using the Arrhenius formula. The results indicated that the evolution of microstructure included the dynamic recrystallization (DRX) of the Zn matrix, the spheroidization of the Mg_2_Zn_11_ phase, and breaking of the FeZn_13_ phase. The subgrains observed within the deformed grain resulted mainly from continuous dynamic recrystallization (CDRX). The precipitated FeZn_13_ grains overlapped with the precipitated MgZn_2_ from the matrix, thus forming a spine-like structure at the phase interface. After compression, the alloy possessed a strong basal texture. Affected by the change of Zn twins, textural strength decreased at first and then increased as the deformation temperature rose. There was only a small unstable region in the processing map, indicating that the alloy exhibited good machinability.

## 1. Introduction

Zinc, one of the essential trace elements inside the human body, is the second most transitional metal element in human body tissues. It participates in the formation of various coenzymes and plays a vital role in body growth, intellectual development and the maintenance of immune function [1]. In recent years, there has been research conducted to demonstrate that zinc and zinc alloys have excellent biocompatibility. Therefore, as the source of a variety of biodegradable implant materials, zinc alloys are recommended by a large majority of scientific researchers [2,3]. However, as-cast pure zinc shows poor mechanical properties and low plasticity, which restricts it from meeting the requirements of some implantable materials, such as stents and bone nails [4]. Thus, it is absolutely necessary to carry out alloying treatment and plastic deformation for the improvement of zinc’s mechanical properties. Differing from the elements added in the creation of zinc alloy structural materials, elements which are toxic (or slightly toxic) to the human body should be discounted from the primary alloying element. Fe, Mg and other elements with excellent biocompatibility are the preferred choice for zinc alloying elements [5,6,7,8,9,10,11]. Most of them tend to form intermetallic compounds with Zn. Though these brittle and hard second phases play a crucial role in increasing the strength of the alloy, they also contribute to increased brittleness [9,12]. Hot deformation is an effective solution to improving plasticity and further increasing strength. As different hot deformation processes endow alloys with different properties, it is essential to analyze the hot deformation behavior of zinc alloys when determining the optimum deformation parameters.

At present, investigation into the hot deformation behavior of zinc alloys focuses mainly on Zn-Cu [13] and Zn-Al [14,15] structural alloy materials. Xu Xiaoqing et al. [13] explored the hot deformation behavior of Zn-0.8Cu-0.3Ti alloy. At a strain rate of 0.01~10 s^−1^ and a temperature between 280 and 350 °C, the cause of softening was identified as dynamic recrystallization. Wu et al. [14] also examined the thermal deformation behavior of Zn10AlCu2 alloy, which led to a judgment that the degree of dynamic recrystallization was higher with temperature rises when the temperature fell below 270 °C. However, that study failed to provide effective guidance on the development of plastic processing techniques for biodegradable alloys. There is still no in-depth research carried out on the flow stress and deformation characteristics of biodegradable zinc-based alloys during the process of high-temperature plastic deformation. Developers require different parameter settings for hot deformation processing (extrusion, rolling, etc.) of degradable zinc alloys. Chao Shen et al. [16] heated Zn-1.2Mg alloy at 250 °C for 2 h, before its extrusion at a temperature of 250 °C and an extrusion ratio of 36:1. E. Mostasd et al. [17] pre-annealed Zn-Mg (0–3 wt.%) at 350 °C for 48 h, prior to its extrusion at 250 °C with an extrusion ratio of 6:1. Song Lin et al. [18] extruded Zn-0.02Mg-0.02Cu at 180 °C at an extrusion ratio of 16:1. Zibo Tang et al. [19] annealed Zn-xCu (1–4 wt.%) at 360 °C for 8 h, before its extrusion at 280 °C with an extrusion ratio of 9:1. Rui Yue et al. [20] extruded Zn-3Cu-xFe (0–1 wt.%)—at a temperature of 180 °C and an extrusion ratio of about 18:1 for 45 min—into a rod. These plentiful, different thermal deformation parameters played no positive role in giving readers an effective reference standard for Zn-Fe-Mg alloys. Therefore, it was imperative that research was undertaken to determine the thermal deformation parameters of Zn-Fe-Mg alloys. Thermal simulation experiments are a commonly used method to reveal the flow stress behavior of zinc alloys. In addition, the construction of a constitutive model is usually preferred to characterize the relationship between the flow stress and thermal deformation parameters at the time of isothermal compression (e.g., strain rate, temperature and equivalent strain).

In this paper, an investigation was conducted into the hot compression behavior of Zn-1Fe-1Mg alloy using a Gleeble-3500 thermo-simulation test machine at a temperature ranging from 235 °C to 340 °C and a strain rate ranging from 10^−2^ s^−1^ to 10 s^−1^. The thermal deformation constitutive model was constructed, the thermal processing diagram was drawn, and the impact of process parameters on the evolution of microstructure and texture was analyzed in detail.

## 2. Materials and Methods

The experimental material was a gravity-cast as-cast Zn-1Fe-1Mg alloy. The alloy ingot size was Φ100 mm × 150 mm; the chemical composition of the alloy ingot for isothermal compression is shown in Table 1. Isothermal compression samples sized Φ10 mm × 15 mm were machined from the bottom of the alloy ingot. The microstructure and quantitative elemental analyses were observed using a field emission scanning electron microscopy (FESEM, JSM-7610F, Mitaka City, Tokyo, Japan). The phase composition of the alloy was analyzed using an X-ray diffraction analyzer (XRD) (XRD-7000, SHIMADZU, Kyoto, Japan) with Cu as the target analyzed. The scanning range was 10–90°, and 5° per minute. Plasma-optical emission spectrometry (ICP-OES, Agilent 725) (Agilent, Santa Clara, CA, USA) was used to analyze the actual proportion of elements in the alloy, as shown in Table 1. Before carrying out the isothermal hot compression experiment, the alloy needed to be homogenized (incubated at 340 °C for 4 h) to soften the structures and eliminate structural defects and segregation.

The isothermal hot compression experiment was carried out on the gleeble-3500D thermomechanical simulator (DSI, St. Paul, MN, USA) at temperatures from 235 °C to 340 °C, and strain rates ranging from 0.01 s^−1^ to 10 s^−1^; the dependent variable was 60%. Prior to isothermal compression, each test sample was heated to the object temperature at a heating rate of 20 °C/s and kept there for 3 min. In order to reduce the interface friction between the alloy and the mold in the thermal deformation experiment, a thin graphite sheet stained with lubricating liquid (machine oil) was placed on the contact surface of the alloy and the mold. After the compression was completed, the compressed sample was quickly quenched in water.

The compressed samples were cut parallel to the compression direction by a wire-electrode cutter, and the sample section was first mechanically polished, then electropolished in a mixed acid solution of 50% phosphoric acid and 50% C2H5OH. Next, the grain structure was characterized by electron backscatter diffraction (EBSD) in JSM-7900F FESEM (Jeol, Mitaka City, Tokyo, Japan). EDAX™ software (OIM analysis 6.2, EDAX, San Diego, CA, USA) was used to process the collected data of EBSD. The average grain size was evaluated using the line intercept method. Low-angle boundaries (LABs) with orientation errors of 2 to 15 are depicted as red lines in the EBSD diagram, while high-angle boundaries (HABs) with orientation errors greater than 15 are indicated by blue lines.

## 3. Results

### 3.1. Flow Stress Behavior

**Figure 1:**
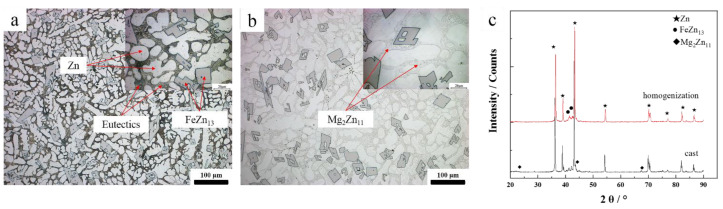
Optical image showing microstructure and XRD patterns of alloy: (**a**) cast alloy, (**b**) homogenized alloy, (**c**) XRD pattern.

Figure 1 shows the microstructure and XRD patterns of the specimens for hot compression, which contained Zn dendrite matrix phase, Zn + Mg_2_Zn_11_ eutectic, and FeZn_13_ needle phase. The Zn grain size of the alloy was 22 μm. From the Zn-Fe and Zn-Mg binary phase diagrams, it can be seen that the solid solubility of Fe and Mg in Zn was very low (0.1 wt.% and 0.02 wt.%, respectively at room temperature) and there was no intermetallic compound between Mg and Fe. In previous studies [21], it was found that the as-cast alloy was composed of Zn matrix, FeZn_13_ prismatic phase and a eutectic phase composed of Mg_2_Zn_11_ + Zn. Figure 1b shows the microstructure of the alloy after homogenization. The Zn in the eutectic structure was dissolved back into the matrix after homogenization, the remaining Mg_2_Zn_11_ phases formed network structures along the grain boundaries, and ports of Mg_2_Zn_11_ phase spheroidized to form many small grains.

**Figure 2:**
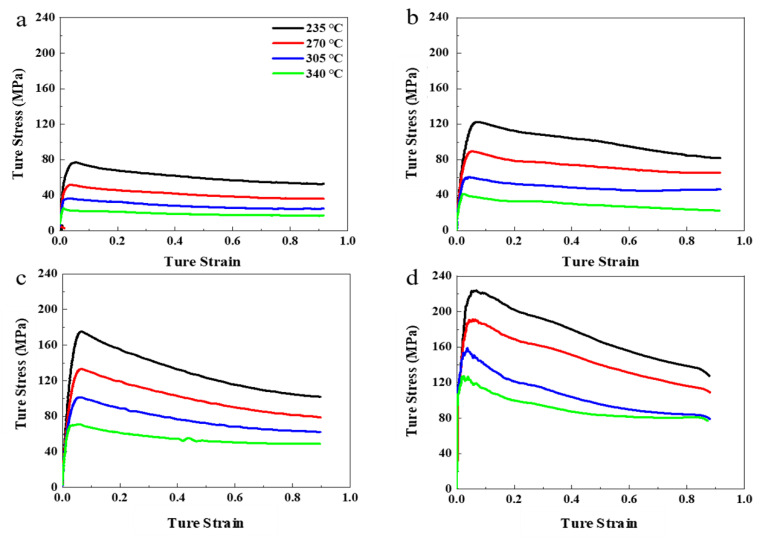
True stress-strain curves of Zn-1Fe-1Mg with various strain rate of (**a**) 10^−2^ s^−1^, (**b**) 10^−1^ s^−1^, (**c**) 1 s^−1^, (**d**) 10 s^−1^.

Because of the short duration of deformation during the deformation process, the deformation heat energy was too difficult to dissipate, which made the sample temperature rise sharply and promoted softening of the grains. Therefore, the stress-strain curve was corrected by adiabatic heating equation, according to the method of Peng et al. [22]. Figure 2 shows the true stress-strain curve of the Zn-1Fe-1Mg alloy in the isothermal unidirectional compression experiment. With the increase of the amount of strain, the alloy’s flow stress displayed an obvious phenomenon—first increasing and then decreasing. This is a characteristic of work hardening-dynamic recrystallization (DRX). In the initial stage of strain, the work hardening effect caused by dislocation diffusion and dislocation winding was far greater than the dynamic recovery and recrystallization effects. As the amount of strain accumulated, the flow stress gradually increased. When the flow stress reached its maximum, the work hardening-dynamic recrystallization reached a dynamic balance. After that, the flow stress gradually reduced, mainly via dynamic recovery and recrystallization.

When the temperature was constant, the flow stress increased with the increase of the strain rate ($\dot{\varepsilon }$). This is because the dislocation density increased with higher strain rates, and so the flow stress increased. At the same strain rate, the flow stress increased as the heating temperature decreased; because the increase in temperature intensified the thermal vibration of atoms inside the grains, it promoted the opening of the alloy slip system and reduced the deformation resistance.

When the strain rate ≥1 s^−1^, one or more waveforms appeared at different temperatures; the higher the strain rate, the more obvious the wave. The higher the strain rate, the earlier the wave appeared—and even sawtooth waveforms appeared, indicating that high strain speed accelerated the nucleation of DRX.

### 3.2. Constitutive Modeling

During the whole unidirectional thermal compression process, the value of flow stress was influenced by the process of work hardening and dynamic recrystallization. It was not a linear control of individual factors, so the Arrhenius equation was introduced to express flow stress behavior of alloy samples [23,24,25]:

(1) $$\dot{\varepsilon }=A(\sinh(\alpha \sigma )){}^{n}\exp(-Q/\mathrm{RT})$$

where $\dot{\varepsilon }$ is the strain rate (s^−1^), σ (MPa) is the flow stress, T (K) is the absolute temperature, Q (KJ·mol^−1^) is the deformation activation energy, R (8.314 J·mol^−1^·K^−1^) is the gas constant.

Introduce Zener–Hollomon parameters [25,26] here:

(2) $$Z\;=\;\dot{\varepsilon }\;\exp(Q/\mathrm{RT})$$

By combining with Equations (1) and (2):

Z = A(sinh(ασ))^n^ (3)

Equation (1) is the flow stress model in the entire stress range. By expanding the Taylor series of Equation (1), two flow stress models with different stress levels can be obtained.

Low stress level:

(4) $$\dot{\varepsilon }=A_{1}\;\sigma^{n1},\;\mathrm{if}\;\alpha \sigma \;<\;0.8$$

High stress level:

(5) $$\dot{\varepsilon }=A_{2}\;\exp(\beta \sigma ),\;\mathrm{if}\;\alpha \sigma \;<\;1.2$$

where A, A1, A2, n1, n, b and a are constants, A is the structure factor, n is the stress exponent, α and β(MPa^−1^) are stress scale parameters (the parameter α can be defined α= β/n1). Considering the effect of strain on rheological stress, it is assumed that A, α, n, and Q are all related to the dependent variable.

The natural logarithms were taken on both sides of Equations (4) and (5) to facilitate the calculation of the material constants, as shown in Equations (6) and (7).

(6) $$\mathrm{Ln}\dot{\varepsilon }=\;{\mathrm{LnA}}_{1}\;+\;n1\mathrm{Ln}\sigma$$

(7) $$\mathrm{Ln}\dot{\varepsilon }=\;{\mathrm{LnA}}_{2}\;+\;\beta \sigma$$

**Figure 3:**
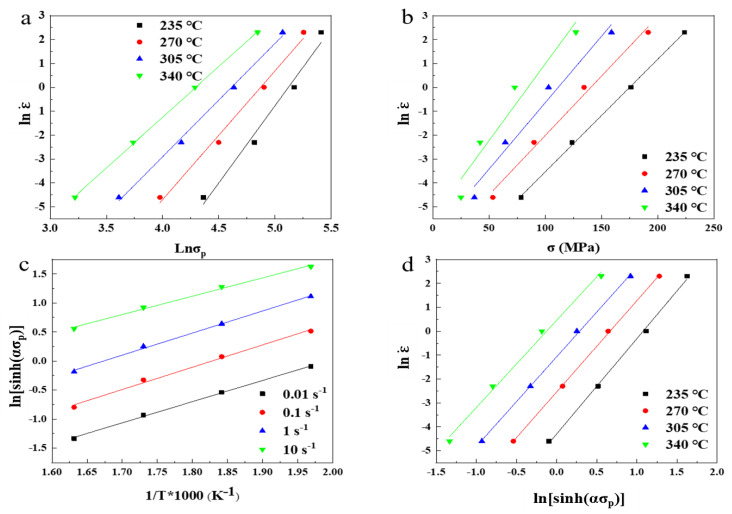
Relationships between (**a**) ln$\dot{\varepsilon }$-lnσ, (**b**) ln$\dot{\varepsilon }$-σ,(**c**) ln(sinh(ασ))-1000/T, and (**d**) ln$\dot{\varepsilon }$-ln(sinh(ασ)).

The relationships between lnσ, σ, ln[sinh(ασ)] and ln$\dot{\varepsilon }$ are plotted in Figure 3 according to Equations (6) and (7). A linear fitting algorithm was applied to measure the value of material constants. The parameters n1, β, α were 5.20799, 0.05419, 0.010404, respectively.

The natural logarithm was introduced to both sides of Equation (1), as shown in Equation (8).

(8) $$\mathrm{Ln}\dot{\varepsilon }\;=\;\mathrm{LnA}\;+\;\mathrm{nLn}(\sin h(a\sigma ))\;-\;Q/\mathrm{RT}$$

**Figure 4:**
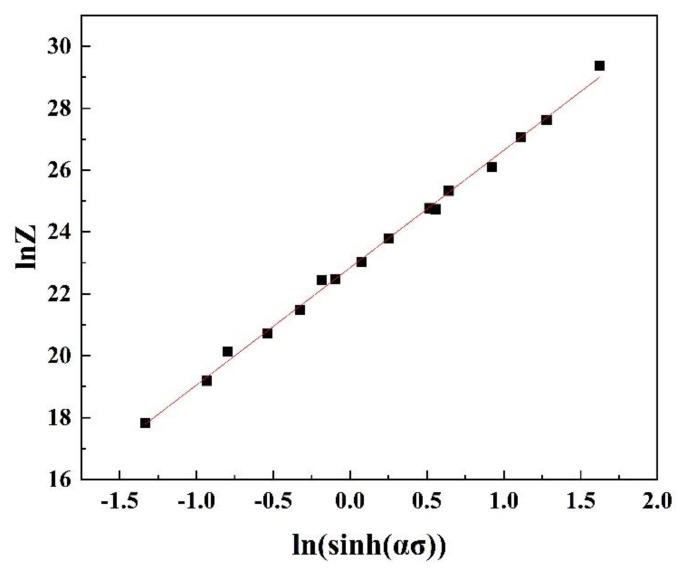
The consistency of experimental and predicted flow stress data using hyperbolic sine equation.

The relationship between 1/T × 1000, ln(sinh (ασ)) and ln$\dot{\varepsilon }$ are plotted in Figure 4 according to Equation (8). We linearly fit the points in the figure; the slope of the straight line was calculated to obtain Q and n values, and the value of A was calculated according to the intercept calculation. The values of Q, n, A were 114.32595 kJ, 3.80256, 8.33 ∗ 10^9^ respectively. The activation energy was 114.33 kJ·mol^−1^, which is higher than the value 95.51 kJ·mol^−1^ of ZA27 reported by Li et al. [23], and lower than the value 150.44 kJ·mol^−1^ of (CNT-Al)/ZA27 composites reported by Liu et al. [15].

At this point all the parameters were obtained and the resulting constitutive equation was:

(9) $$\dot{\varepsilon }\;=\;8.33\;\times \;10^{9}\;\times \;(\sin h(0.0104\sigma ))3.803\exp(-114326/(\mathrm{RT}))$$

The constitutive equation was verified by Zener–Hollomon factor. We substituted Equation (2) into Equation (1), and then took the logarithm on both sides of equation, obtaining Equation (10):

LnZ = LnA + nLn(sinh(ασ))(10)

According to the above Equation (10), we made a relationship graph about LnZ-Ln (sinh(ασ)), and linearly fit the points in the graph, demonstrated in Figure 4. The R-squared value of linear correlation was 0.996, which proved that the Arrhenius formula accurately demonstrated the flow stress behavior of the Zn-1Fe-1Mg alloy.

Dynamic material modeling (DMM) can reflect the internal microstructure changes of the material deformed under various deformation temperatures and strain rates, and the processing performance of the material can be evaluated [27,28]. The processing map of Zn-1Fe-1Mg alloy was built on the basis of DMM. The total power dissipation P was composed of the power dissipated by plastic deformation (G) and the power dissipated by microstructure transformation (J):

(11) $$P\;=\;\sigma \dot{\varepsilon \;}=\;G\;+\;J\;=\;\int_{0}^{\dot{\varepsilon }}\sigma d\dot{\varepsilon }\;+\;\int_{0}^{\sigma }\dot{\varepsilon }\;d\sigma$$

When the deformation temperature was constant, the relationship between flow stress and strain rate were the following Equation (12):

(12) $$\Sigma \;=\;K\dot{\varepsilon }{}^{m}$$

where K is a constant, m is the sensitivity factor of strain rate, and the following is its calculation equation:

(13) $$M\;=\;\partial (\ln\sigma )/\partial (\ln\dot{\varepsilon })$$

The dimensionless power dissipation factor η can be expressed by the strain rate sensitivity factor m, which varies with temperature and strain rate to form a power dissipation graph. The power dissipation diagram represents the power dissipation when the microstructure of the material changes, and is called the microstructure trajectory [17]. Its definition is as follows:

H = J/J_max_ = 2m/(1 + m) (14)

The instability judgment basis of DMM was established by Prasad; the equation is:

(15) $$\xi (\dot{\varepsilon })=(\partial \ln(m/(m+1)))/(\partial \ln\varepsilon )+m$$

When ξ($\dot{\varepsilon }$) is less than 0, it can be judged that the material has rheological instability.

**Figure 5:**
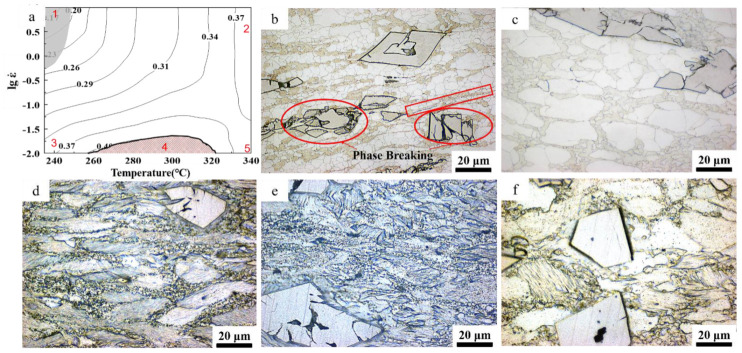
Processing map and microstructure diagram of corresponding position: (**a**) processing map; (**b**) microstructure of alloy at position 1; (**c**) microstructure of alloy at 340 °C and 0.01 s^−1^; (**d**) the microstructure of alloy at position 3; (**e**) the microstructure of alloy at position 4; (**f**) the microstructure of the alloy at position 5.

The hot processing map was the combination of the instability map and the power dissipation map. Figure 5a exhibits the processing map of the Zn-1Fe-1Mg under the compression quantity of 60%. Its horizontal axis and vertical axis were, respectively, the strain rate range (10^−2^ s^−1^~10 s^−1^) and the temperature range (235~340 °C). The power dissipation efficiency is represented by contour lines. The number represents the percentage of power dissipation efficiency. The shaded area in the processed map is the instability domain, and is represented by x < 0. It was easily proved from the processing map that η increased as the test temperature increased or the strain rate decreased. The power dissipation factor in more than two-thirds of the region exceeded 0.30, indicating that the alloy had good workability and a high-volume fraction of recrystallized grain transformation. There was only a small region of instability in the processing diagram, which had a temperature range of 235~250 °C and a strain rate of 0.5 s^−1^~10 s^−1^. Processing under an instability zone will cause defects in the alloy structure and reduce mechanical properties, so this area should be avoided during processing.

**Figure 6:**
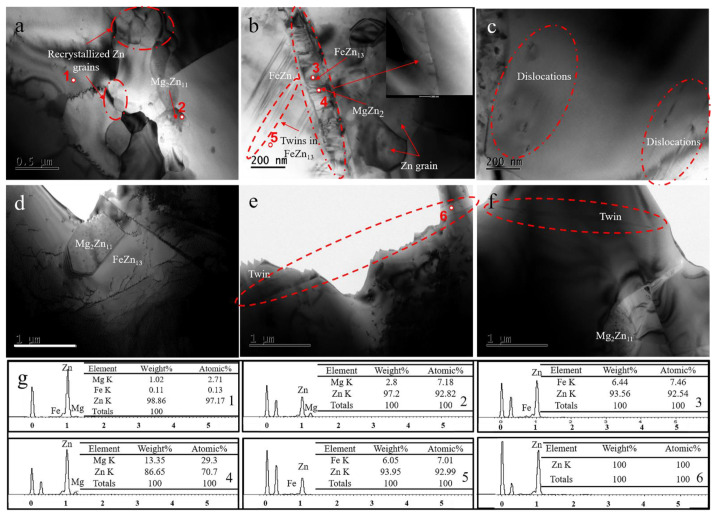
TEM images of the microstructure in unidirectional compression alloys: (**a**,**b**,**c**) 235 °C,10 s^−1^; (**d**,**e**,**f**) 340 °C, 0.01 s^−1^; (**g**) EDS analysis of the corresponding position to the number.

Figure 5b–f shows the microstructure of the alloy sample under isothermal compression. Compared with the homogenized alloy sample, the microstructure underwent significant changes. The grains were compressed and elongated, and dynamic recrystallized grains appeared inside the grains; thus, the grain size was significantly smaller than that after homogenization. The Mg_2_Zn_11_ phase was further broken into small spheres and distributed in a necklace-like shape along the original grain boundary. The red frame in Figure 6b shows that several Mg_2_Zn_11_ chains were gathered together, and many small DRX grains appeared around them. This local shaping flow behavior may cause plastic instability. Part of the stored energy was released by the fragmentation of the FeZn_13_ phase, which can also lead to processing failure. When decreases in the strain rate or increases in the temperature, the η increased. This may have been due to more dynamic recrystallization at the grain boundaries.

From the microstructure map corresponding to the thermal processing map, it was determined that although the 2, 3, and 5 positions had similar power dissipation factors, their microstructures differed significantly. Interestingly, at low strain rates, in addition to dynamic recrystallization, there was also a deformed twin structure in the alloy. Similar results were shown in Xu’s research on Zn-8Cu-0.3Ti [13,29]. Although zinc alloy has a close-packed hexagonal structure, its c/a = 1.86, which is higher than the ideal 1.73. So, in the process of plastic deformation, twinning occurred when the force-bearing base surface was deflected to the parallel hard orientation. The twinning caused the crystal orientation in the twinning region to deviate from the sliding hard phase, which promotes continued slippage and improves plastic deformability. Sliding reached the hard orientation position more quickly at high temperatures or low strain rates, causing twins to form quickly.

### 3.3. Microstructural Evolution

#### 3.3.1. TEM Analysis of Deformation Alloys

To deeply explore the effects of hot deformation behavior on the microstructure of samples, thermal simulation conditions of 235 °C, 10 s^−1^ and 340 °C, 0.01 s^−1^ across two samples were observed by TEM, as shown in Figure 6. It can be clearly observed from Figure 6 that the increase in temperature (or the decrease in strain rate) promoted the growth of Zn crystal grains and Mg_2_Zn_11_ grains. As for the FeZn_13_ phase, it showed thermal stability at 235~340 °C. Thus, its size did not change.

During the experiment, dislocation sliding may have been the main deformation mechanism. FeZn_13_ and Mg_2_Zn_11_ are hard-brittle phases, and their hardness is higher than the Zn matrix (Mg_2_Zn_11_ is 110 ± 5 HV, which was obtained by measuring the eutectic in cast Zn-3Mg, FeZn_13_ is 243 ± 8 HV, Matrix is 76.1 HV ± 2 HV), leading to deformation incompatibility at the phase interface. Stress concentration happened near the phase interface, which caused the sliding of dislocations. The entanglement of dislocations accelerated the nucleation of dynamic recrystallization at the phase boundaries. Under the compression conditions of low temperature and high strain rate, residual parallel straight dislocations could still be observed inside the grains (see Figure 6c). This is because the thermal activation energy in the system was low and the deformation time was extremely short; in that situation, dislocations could not be completely eliminated by dynamic recrystallization. At same time, residual stress was high, which caused the retention of more deformed grains and subcrystals, which will be discussed in the next section. FeZn_13_ is a monoclinic structure with only a few slip systems, and convex-like twinning seemed to occurs during the deformation process, as shown in Figure 6b. The interface between the FeZn_13_ phase and the Zn crystal grains was a stress concentration region. The small-sized FeZn_13_ phase precipitated here alternately overlapped with the MgZn_2_ precipitated from the matrix to form a spine-like structure. When the test temperature was high, the intense thermal motion of atoms inside the crystal grains promoted the nucleation and growth of DRX, so the DRX grain boundaries seemed to be invisible (see Figure 6d). At the same time, the high temperature seemed to promote the formation of twins in the crystal grains, as shown in Figure 6e,f.

#### 3.3.2. Influence of Compression Parameters on Grain Structure

##### Microstructure Evolution with the Changing of Temperature

**Figure 7:**
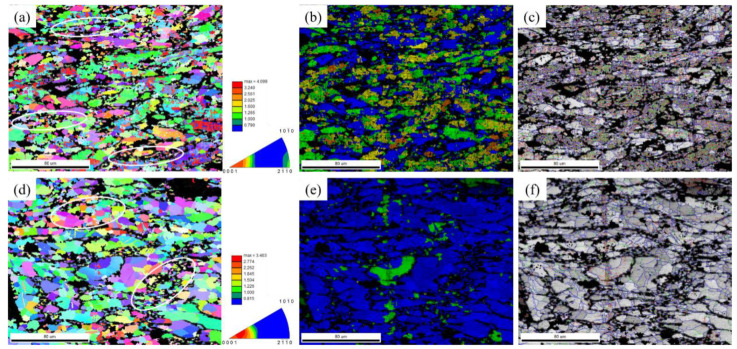

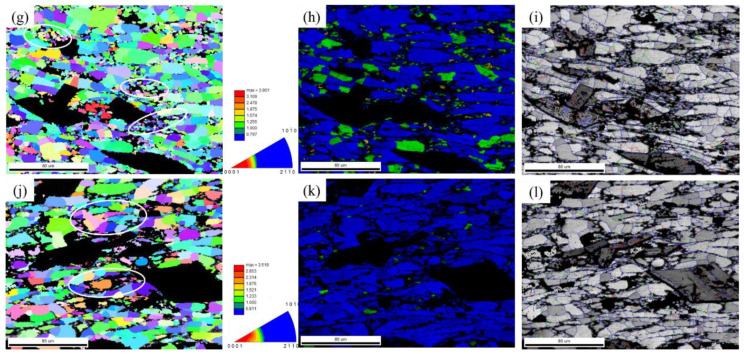
Electron backscatter diffraction (EBSD) of isothermal compression alloy samples at a strain rate of 10 s^−1^: (**a**) inverse pole figure map at 235 °C; (**b**) crystal grain orientation map at 235 °C; (**c**) grain boundary map at 235 °C; (**d**) inverse pole figure map at 270 °C; (**e**) crystal grain orientation map at 270 °C; (**f**) grain boundaries at 270 °C; (**g**) inverse pole figure map at 305 °C; (**h**) crystal grain orientation map at 305 °C; (**i**) grain boundaries map at 305 °C; (**j**) inverse pole figure at 340 °C; (**k**) crystal grain orientation map at 340 °C; (**l**) grain boundaries map at 340 °C.

The EBSD maps of alloy samples (after unidirectional compression at temperatures of 235~340 °C with a strain rate of 10 s^−1^) are shown in Figure 7. In the grain distribution diagram, blue represents recrystallized grains, green and yellow represent subcrystalline grains, and orange and yellow represent deformed structures. After electrolytic polishing, there was a height difference between these second phases and Zn crystal grains, so an undefined area appears in the corresponding position on the EBSD maps; it appears black.

At a high strain rate of 10 s^−1^, the transition of dynamic recrystallization was not complete. Hence, the microstructure was dominated by recrystallized grains with a few sub-grains and deformed structures. These second phases were in a hard orientation with adjacent crystal grains, hindering the dislocation slip of the zinc crystal grains and becoming a larger deformation energy region. This caused dislocations to easily accumulate near the boundary of the second phase and cause stress concentration. Compared with inside the grain, this place had higher energy accumulations, where dynamic recrystallization was more likely to occur. The nucleation and growth of DRX were controlled by the continuous dynamic recrystallization mechanism (CDRX). The main feature of CDRX is the appearance of subgrain boundaries within grains—followed by a gradual transformation to large-angle grain boundaries (see Figure 7b,e,h,k).

When the test temperature was 235 °C, it was observed that the microstructure was dominated by the deformed structure and subcrystalline grains, and that the proportion of recrystallized grains was relatively low. With the temperature increasing, the recrystallized grains gradually grew, the volume fraction increased, and the deformed structure and subcrystals gradually decreased. One possible reason is that—under the condition of a strain rate of 10 s^−1^—the strain energy was very high, but the rate of dynamic recrystallization formation and growth at low temperatures was slow, and a large amount of stress gradually accumulated quickly and could not be released. Thus, it was stored in deformed grains and subcrystals. As the temperature increased, the recrystallization thermodynamics increased, as did the power dissipation factor (from 0.15 to 0.38), indicating that the proportion of energy used for phase change similarly increased. Therefore, the rate of DRX increased rapidly, which also increased the size of recrystallized grains. At high strain rates, the c-axis of the alloy Zn grains after thermal compression was perpendicular to the compression direction, which made the alloy matrix exhibit a strong basal texture. As the temperature gradually decreased, the texture orientation factor became larger and larger.

##### The Effect of Strain Rate Change on the Grain Structure

**Figure 8:**
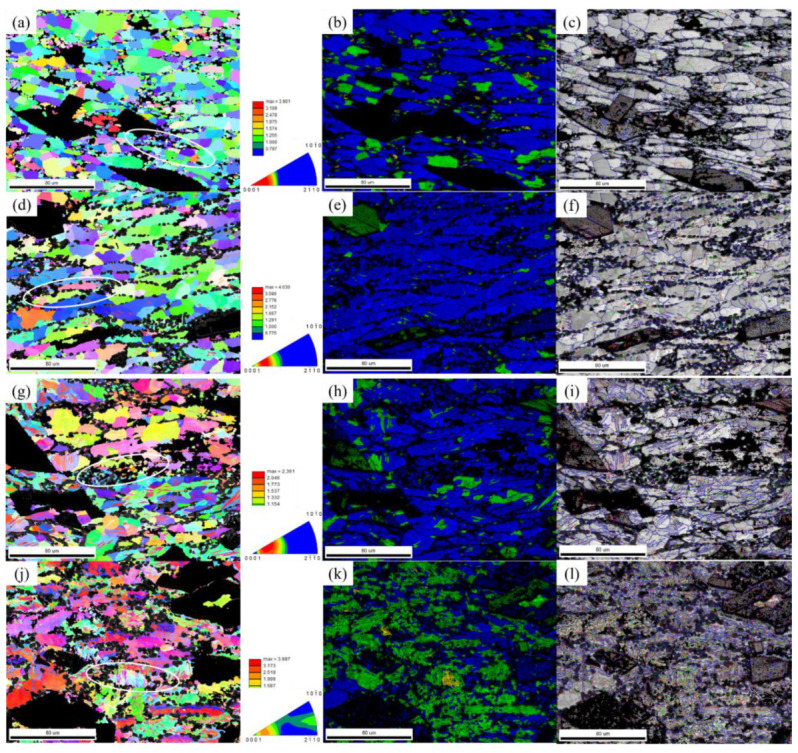
EBSD images of isothermal compressed alloy samples at a temperature of 305 °C: (**a**) inverse pole figure at 10 s^−1^; (**b**) crystal grain orientation map at 10 s^−1^; (**c**) grain boundaries map at 10 s^−1^; (**d**) inverse pole figure at 1 s^−1^; (**e**) crystal grain orientation map at 1 s^−1^; (**f**) grain boundaries at 1 s^−1^; (**g**) inverse pole figure at 0.1 s^−1^; (**h**) crystal grain orientation map at 0.1 s^−1^; (**i**) grain boundaries map at 0.1 s^−1^; (**j**) inverse pole figure at 0.01 s^−1^; (**k**) crystal grain orientation map at 0.01 s^−1^; (**l**) grain boundaries map at 0.01 s^−1^.

Figure 8 shows EBSD images of the alloy samples, hot compressed at a temperature of 305 °C with different strain rates (10^−2^ s^−1^~10 s^−1^). For one compressed at a strain rate of 10 s^−1^, the high strain rate led to more entangled dislocations and a high stress concentration. Due to the short deformation time, the recrystallized grains produced were small. A small number of twins were produced at the same time, and there were still partially deformed grains. When the strain rate decreased, the size of the recrystallized grains gradually increased, the number of twins gradually decreased, and the deformed grains were gradually eliminated. When the strain rate is slow, the dynamic softening time increases significantly; this makes the migration of grain boundaries easier, and the deformed structure in the alloy is greatly reduced. When the alloy strain rate was 0.01 s^−1^ for hot compression, there was little dislocation accumulation and the dislocation density was low. Therefore, it may not have been able to meet the kinetic and thermodynamic requirements of dynamic recrystallization. This makes it difficult to transform subgrain boundaries to large-angle grain boundaries. The stress can be released by generating twins, but twins cannot completely eliminate alloy deformation, leaving many subcrystalline grains. In the strain rate range of 0.01 s^−1^ to 10 s^−1^, the c-axis of the alloy grains gradually developed perpendicular to the direction of compression, thus forming a strong basal surface texture. With the gradual increase of the strain rate, the strength of the texture increased, and the direction moved closer and closer to the (0001) plane.

## 4. Conclusions

In this work, the flow stress behavior and microstructural evolution of a Zn-1Fe-1Mg alloy isothermally compressed under various ranges of temperature and strain rate was examined. The major conclusions of this study can be summarized as follows:

1. In the process of isothermal compression, as the amount of strain accumulates, the flow stress quickly reaches a peak value and then slowly decreases. The peak stress gradually increases as the compression temperature decreases (or as the strain rate increases). The constitutive equation for hot deformation is $\dot{\varepsilon \;}$ = 8.33 × 10^9^ × (sinh(0.0104σ))^3.803^exp(−114326/(RT)). There is a satisfactory consistency between the expected flow stress and the experimental flow stress during the process of hot compression.
2. Power dissipation is affected by both strain rate and heating temperature. At low temperatures and high strain rates (235~250 °C, 10 s^−1^~0.5 s^−1^), the power dissipation factor is very low, and there is a small area of processing instability. As the temperature increases and the strain rate decreases, the power dissipation factor gradually increases, reaching its highest value (0.40) at the temperature of 305 °C and the strain rate of 0.01 s^−1^.
3. The nucleation and growth of DRX are controlled by the mechanism of CDRX. The increase in temperature and the decrease in strain rate reduce the deformed structure and subcrystals inside the grains, but promote the formation of intragranular twins.

The recrystallized nanosized FeZn_13_ phase appeared, and the MgZn_2_ phase precipitated from the matrix formed a spine-like structure with it. As the temperature increased and the strain rate decreased, the recrystallized grains gradually grew up, increasing the intensity of the basal texture.

## Figures and Tables

**Table 1:** Chemical composition of Zn-1Fe-1Mg alloy (wt.%).

Zn	Mg	Fe	Cu	Si	Ni
Balance	1.19	0.97	0.02	0.02	0.02

## Data Availability

No new data were created or analyzed in this study.
